# Overexpression of L-Type Amino Acid Transporter 1 (LAT1) and 2 (LAT2): Novel Markers of Neuroendocrine Tumors

**DOI:** 10.1371/journal.pone.0156044

**Published:** 2016-05-25

**Authors:** Susi Barollo, Loris Bertazza, Sara Watutantrige-Fernando, Simona Censi, Elisabetta Cavedon, Francesca Galuppini, Gianmaria Pennelli, Ambrogio Fassina, Marilisa Citton, Beatrice Rubin, Raffaele Pezzani, Clara Benna, Giuseppe Opocher, Maurizio Iacobone, Caterina Mian

**Affiliations:** 1 Endocrinology Unit, Department of Medicine, University-Hospital of Padua, Padua, Italy; 2 II Pathology Unit, Department of Medicine, University-Hospital of Padua, Padua, Italy; 3 Surgical Pathology Unit, Department of Surgery, Oncology and Gastroenterology Sciences, University-Hospital of Padua, Padua, Italy; 4 Familial Cancer Clinic and Oncoendocrinology, Veneto Institute of Oncology, Padova, Italy; University of Bern, SWITZERLAND

## Abstract

**Background:**

6-18F-fluoro-L-3,4-dihydroxyphenylalanine (^18^F-FDOPA) PET is a useful tool in the clinical management of pheochromocytoma (PHEO) and medullary thyroid carcinoma (MTC). ^18^F-FDOPA is a large neutral amino acid biochemically resembling endogenous L-DOPA and taken up by the L-type amino acid transporters (LAT1 and LAT2). This study was conducted to examine the expression of the LAT system in PHEO and MTC.

**Methods:**

Real-time PCR and Western blot analyses were used to assess *LAT1* and *LAT2* gene and protein expression in 32 PHEO, 38 MTC, 16 normal adrenal medulla and 15 normal thyroid tissue samples. Immunohistochemistry method was applied to identify the proteins’ subcellular localization.

**Results:**

*LAT1* and *LAT2* were overexpressed in both PHEO and MTC by comparison with normal tissues. *LAT1* presented a stronger induction than *LAT2*, and their greater expression was more evident in PHEO (15.1- and 4.1-fold increases, respectively) than in MTC (9.9- and 4.1-fold increases, respectively). Furthermore we found a good correlation between *LAT1/2* and *GLUT1* expression levels. A positive correlation was also found between urinary noradrenaline and adrenaline levels and *LAT1* gene expression in PHEO. The increased expression of LAT1 is also confirmed at the protein level, in both PHEO and MTC, with a strong cytoplasmic localization.

**Conclusions:**

The present study is the first to provide experimental evidence of the overexpression in some NET cancers (such as PHEO or MTC) of L-type amino acid transporters, and the LAT1 isoform in particular, giving the molecular basis to explain the increase of the DOPA uptake seen in such tumor cells.

## Introduction

Functional imaging is an important step in the diagnostic work-up of patients with tumors of neuroendocrine origin, such as pheochromocytoma (PHEO) and medullary thyroid carcinoma (MTC). Such radiological procedures can contribute to confirm a diagnosis of neuroendocrine tumor (NET), stage the disease on presentation, or restage after treatment, identify patients amenable to targeted radionuclide therapies, and reveal the metabolic response to such clinical approaches [1]. In the past, the most extensive functional imaging option involved scintigraphy with ^123^I-metaiodobenzylguanidine (MIBG) or ^18^F-FDG, but 6-18F-fluoro-L-3,4-dihydroxyphenylalanine (^18^F-FDOPA) scintigraphy has emerged more recently as a useful tool in the clinical management of NET [2,3].

The rationale for using ^18^F-FDOPA scintigraphy is based on the fact that NET are able to take up decarboxylate and store amino acids and their biogenic amines, such as L-DOPA [4]. L-DOPA is an amino acid containing two hydroxyl groups on the phenol ring and a precursor along the catecholamine synthesis pathway. L-DOPA is taken up in the cytoplasm of neuroendocrine cells by a L-type amino acid transporter (LAT) system; it is metabolized to dopamine, which is trapped in secretory vesicles by the vesicular monoamine transporters (VMAT) types 1 and 2, and further metabolized to noradrenaline and adrenaline. For imaging purposes, L-DOPA can be radiolabeled with the positron emitter isotope 18F in the sixth position, thus forming ^18^F-FDOPA, which can then be used in PET imaging [5]. ^18^F-FDOPA is a large neutral amino acid that shares many structural similarities with natural L-DOPA. It moves into the same catecholamine metabolic pathway as its natural counterpart, mirroring its endogenous kinetics (both in the brain and peripherally) [6].

Recent studies have identified the two main different isoforms of LAT belonging to the SLC7 transporter gene family, i.e. *LAT1* (or *SLC7A5*) and *LAT2* (or *SLC7A8*). LAT1 and LAT2 are sodium-independent neutral amino acid transporters that are active in renal epithelial cells, brain capillary endothelial cells, and normal and neoplastic neuroendocrine cells [7]. In particular, LAT1 is known to be strongly expressed in many tumor cell lines and in primary human tumors, in which it has been shown to play an essential part in growth and survival [8–10]. For their growth and survival, cancer cells also rely on their glucose metabolism in order to obtain ATP molecules, and this process demands a greater supply of glucose that is mediated mainly by the GLUT1 transmembrane transporters [11], that represents the molecular basis for radiolabeled glucose, used in FDG-PET.

PHEO and MTC are rare NET. PHEO are catecholamine-secreting tumors that usually arise from chromaffin cells of the adrenal medulla; about 15–20% of such tumors derive from extra-adrenal chromaffin tissues (extra-adrenal PHO or paragangliomas) [12]. Approximately 30% of catecholamine-secreting tumors carry a germline mutation (Iacobone et al. 2011). MTC is a rare thyroid cancer (2–4% of cases) deriving from parafollicular C-cells; it may be hereditary in approximately 25% of cases, due to germinal mutations activating the RET proto-oncogene in MEN2 syndrome.

The aims of this study were: 1) to study the LAT system in tissues obtained from patients with PHEO or MTC with a view to elucidating the molecular grounds for using ^18^F-FDOPA in the diagnostic/clinical work-up; 2) to correlate the expression of LAT with that of GLUT1 transporters in the same tissues; and 3) to identify any relationships between LAT expression and patients’ clinical features.

## Materials and Methods

### Patients

The study concerned a consecutive series of 32 patients with PHEO, all but 4 of them sporadic, and all but 2 benign (10 men and 22 women; median age 55, range 25–74 years), and 38 patients with MTC, all but 3 of them sporadic (18 men and 20 women; median age 60, range 10–81 years). Only one of the patients showed both PHEO and MTC.

Normal adrenal medulla tissue samples (n = 16) were obtained during surgical procedures for the purposes of renal transplantation. Normal thyroid tissue samples (n = 15) were obtained from the contralateral thyroid lobe in patients undergoing thyroid surgery for unifocal differentiated thyroid cancers.

All studies were performed in accordance with the guidelines proposed in the Declaration of Helsinki: the local ethical committee (Ethical Committee for the Clinical Experimentation of the Hospital of Padua) approved our study protocol (Ref. 3388) and all patients (including the parent/guardian on behalf of the minor) gave their written informed consent.

### Mutation screening by direct sequencing

At germinal level, all exons of succinate dehydrogenase complex B *(SDHB)*, *SDHD*, and *VHL*, and exons 5, 8, 10, 11 and 13–16 of the *RET*, and *MAX* and *TMEM127*, and exons 2–10 of *MEN-1* were examined by direct sequencing. Somatic mutations of *N-K-H RAS* and *RET* were also analyzed in cases of sporadic MTC.

### RNA extraction and reverse transcription

Each surgical specimen was snap-frozen in liquid nitrogen within 15 minutes of collection and stored at -80°C pending RNA recovery. Total RNA was extracted using the TRIzol reagent lysis buffer (Invitrogen, Life Technologies, Carlsbad, CA) according to the manufacturer's protocol.

### qRT-PCR

A real-time quantitative PCR (qRT-PCR) was performed in an ABI-PRISM 7900HT Sequence Detector (Applied Biosystems, Milan, Italy) using the relative quantification method (2^-ΔΔCt^ method) as previously described [13]. The genes were analyzed using the following TaqMan assays: *SLC7A5* (Hs00185826_m1); *SLC7A8* (Hs00794796_m1); and *GLUT1* (Hs00892681_m1), all from Applied Biosystems. Data were analyzed with the Sequence Detection Software rel. 2.4 (Applied Biosystems), adopting an automatically-set baseline and a fluorescence threshold adjusted to measure quantification cycle (Ct) values. Validation experiments performed using the standard curve method with five serial dilutions of genomic DNA from control subjects showed identical amplification efficiencies (100% ± 10%) calculated according to the formula E = 10^1/-slope^-1 for all assays. Using the 2^-ΔΔCt^ method the data were presented as the fold-change in gene expression normalized by a reference gene and relative to a calibrator sample. As the reference gene in this study we used β-actin (Hs99999903_m1), one of the most commonly used housekeeping genes. A pool of cDNA derived from mixed normal human thyroid and adrenal medulla tissues was used as the calibrator source in our study.

### Western blot analysis

Tumor samples were collected after surgery, frozen immediately in liquid nitrogen, and stored at −80°C. Immunoblot analysis was performed on normal and cancerous frozen thyroid tissue segments, as described elsewhere [14]. Briefly, proteins were separated with SDS/PAGE under reducing condition, in the presence of the S-S reducing agent dithiothreitol (DTT), electroblotted onto nitrocellulose membranes and saturated in 5% non-fat dry milk. Membranes were incubated overnight with the primary antibodies [anti-SLC7A5 (1:1000), Abcam (Cambridge, UK) and anti-β-actin, Sigma-Aldrich (1:5000)] and then incubated with HRP-conjugated secondary antibodies (Jackson ImmunoResearch, Europe). Blots were developed using Pierce ECL Substrate and exposed to CL-XPosure Film (Thermo Scientific, Rockford, US). Films were scanned and band intensity was quantified with ImageJ software 1.44p. To validate the anti-SLC7A5 antibody stain, LI-COR Odyssey Imaging Systems with the specific infrared fluorescence IRDye^®^ secondary antibodies were used (S1 Fig) [15]. All experiments were performed in duplicate.

### Immunohistochemistry

To further validate the immunofluorescence data, immunohistochemistry was performed on formalin-fixed, paraffin-embedded tissue sections 4–6 μm thick from 5 PHEO and 5 MTC cases, using the same anti-LAT1 antibody (1:500). Appropriate positive and negative controls were run concurrently. Two different pathologists (G.P. and F.G.) blindly assessed the findings, describing the intensity of staining as weak, moderate or strong. The subcellular localization of staining was also considered.

### Statistical analysis

Proportions and rates were calculated for categorical variables; means ± standard deviations, or medians and ranges for parametric or non-parametric variables. In the qRT-PCR experiments, groups were compared with the Mann-Whitney or Wilcoxon tests for quantitative variables not distributed normally. Spearman’s rank correlation and regression analyses were used to test the association between mRNA expression and different genes. To correlate the clinical/pathological features and LAT1/2 gene expression in PHEO/MTC, the PHEO/MTC patients in our series were divided into two groups (PHEO/MTC strongly or weakly expressing LAT) based on median receptor expression values, using the chi-square and Fisher’s exact tests as appropriate. The MedCalc for Windows, version 14.7 (MedCalc software, Ostend, Belgium) was used to manage the dataset on our patients and for the statistical analyses. The level of significance was set at p < 0.05 for all tests.

## Results

### *LAT1*, *LAT2* and *GLUT1* gene expression in PHEO

**Fig 1:**
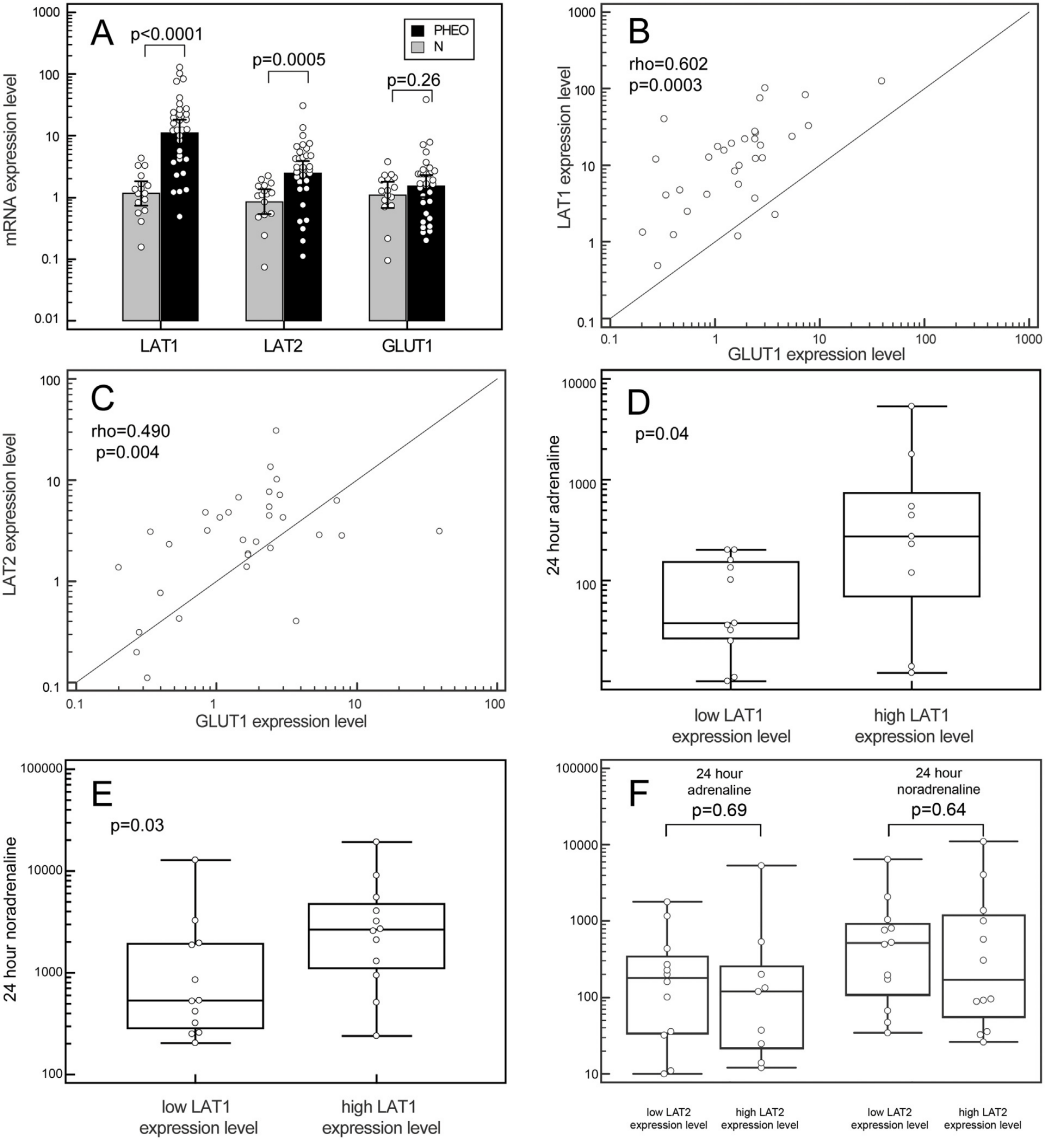
*LAT1*, *LAT2* and *GLUT1* genes expression in PHEO. (**A**) Box plots of relative qPCR gene expression measurements of *LAT1*, *LAT2* and *GLUT1* in PHEO specimens and the relative paired normal tissues. Each value was referred to a pool of normal thyroid tissues that was set to 1. Each dot represents a single sample. (**B**) and (**C**) Associations between the *LAT1* and *GLUT1* (**B**) and the *LAT2* and *GLUT1* (C) gene expressions in PHEO specimens, by Spearman’s rank correlation. (**D**) and (**E**) Box plots representing the correlation between the *LAT1* expression levels in PHEO, dichotomized according to the median value, with the urinary levels of adrenaline (**D**) and noradrenaline (**E**). (**F**) Box plots representing the correlation between LAT2 expression levels in PHEO, dichotomized according to the median value, with the urinary levels of adrenaline (left) and noradrenaline (right). Boxes indicate the range from lower to upper quartile values, with the line inside the box representing the median. The vertical lines mark the highest and lowest value observed within a distance of 1.5 times the inter-quartile range from the bottom and the top of the boxes, respectively.

PHEO specimens showed a significant increase in *LAT1* (p<0.0001) and *LAT2* (p = 0.0005) mRNA levels by comparison with normal adrenal medulla tissues. No statistically significant difference was found in *GLUT1* (p = 0.26) mRNA expression levels between pathological and normal tissues. In particular, the median upregulation was 15.1-fold for *LAT1* (95% CI: 12.54–34.92) and 4.1-fold for *LAT2* (95% CI: 2.43–4.16) (Fig 1A). *LAT1* mRNA was more overexpressed that of *LAT2* in PHEO samples (p = 0.0001), while no differences were found between the two transporters’ expression levels in normal adrenal medulla tissues.

Spearman’s rank correlation coefficients were calculated to investigate the associations between the *LAT1*, *LAT2* and *GLUT1* gene expressions in PHEO. There was a strongly positive relationship between *LAT1* and *GLUT1* expression (rho = 0.602, p = 0.0003) (Fig 1B), and a positive relationship between *LAT2* and *GLUT1* expression (rho = 0.490, p = 0.004) (Fig 1C).

A statistically significant link also emerged between *LAT1* overexpression and high urinary levels of noradrenaline and adrenaline (p = 0.04 and p = 0.03, respectively, Fig 1D and 1E), while this was not apparent for *LAT2* (Fig 1F). Patients’ clinical characteristics are summarized in Table 1.

**Table 1:** Clinical features of patients with pheochromocytoma.

*N°*	*Gender*	*Age at diagnosis (years)*	*24-hour adrenaline(nmol)*	*24-hour noradrenaline(nmol)*	*Size (mm)*	^†^*LAT1 expression level*
1	F	60	200	258	30	low
2	F	71	1178	415	35	low
3	M	53	n.a.	n.a.	45	low
4	F	55	5352	3206	35	high
5	F	60	37.4	538	18	low
6	F	60	160	1879	33	low
7	M	74	32	1960	60	low
8	F	37	n.a.	19188	35	high
9	F	49	1801	2589	35	high
10	F	52	14	9019	35	high
11	F	47	n.a.	240	40	high
12*	F	66	n.a.	1309	25	high
13	F	63	542	2106	35	high
14	M	50	n.a.	n.a.	30	high
15	M	57	273	2698	39	high
16	M	63	n.a.	511	65	high
17* ^¶^	F	37	231	5500	60	high
18	F	55	36	850	95	low
19	F	39	n.a.	n.a.	45	low
20	F	48	n.a.	n.a.	65	high
21	F	55	n.a.	n.a.	45	low
22	F	68	119	4056	47	high
23	F	63	101	319	35	low
24^§^	M	51	200	3266	180	low
25*^§^	M	55	11	12886	60	low
26	F	48	n.a.	n.a.	60	low
27*	F	25	133	203	47	low
28	M	72	441	938	25	high
29	F	63	10	251	22	low
30	M	43	12	n.a.	70	high
31	F	38	n.a.	n.a.	34	high
32	M	34	25	525	70	low

Abbreviations: F, female; M, male; n.a., not available,

*familiar,

^§^malignant,

^¶^MEN2 patient where PHEO and MTC were analyzed simultaneously for LAT1 mRNA expression level (see results section).

^†^ LAT1 mRNA expression divided into high and low levels according to the median value.

**Fig 2:**
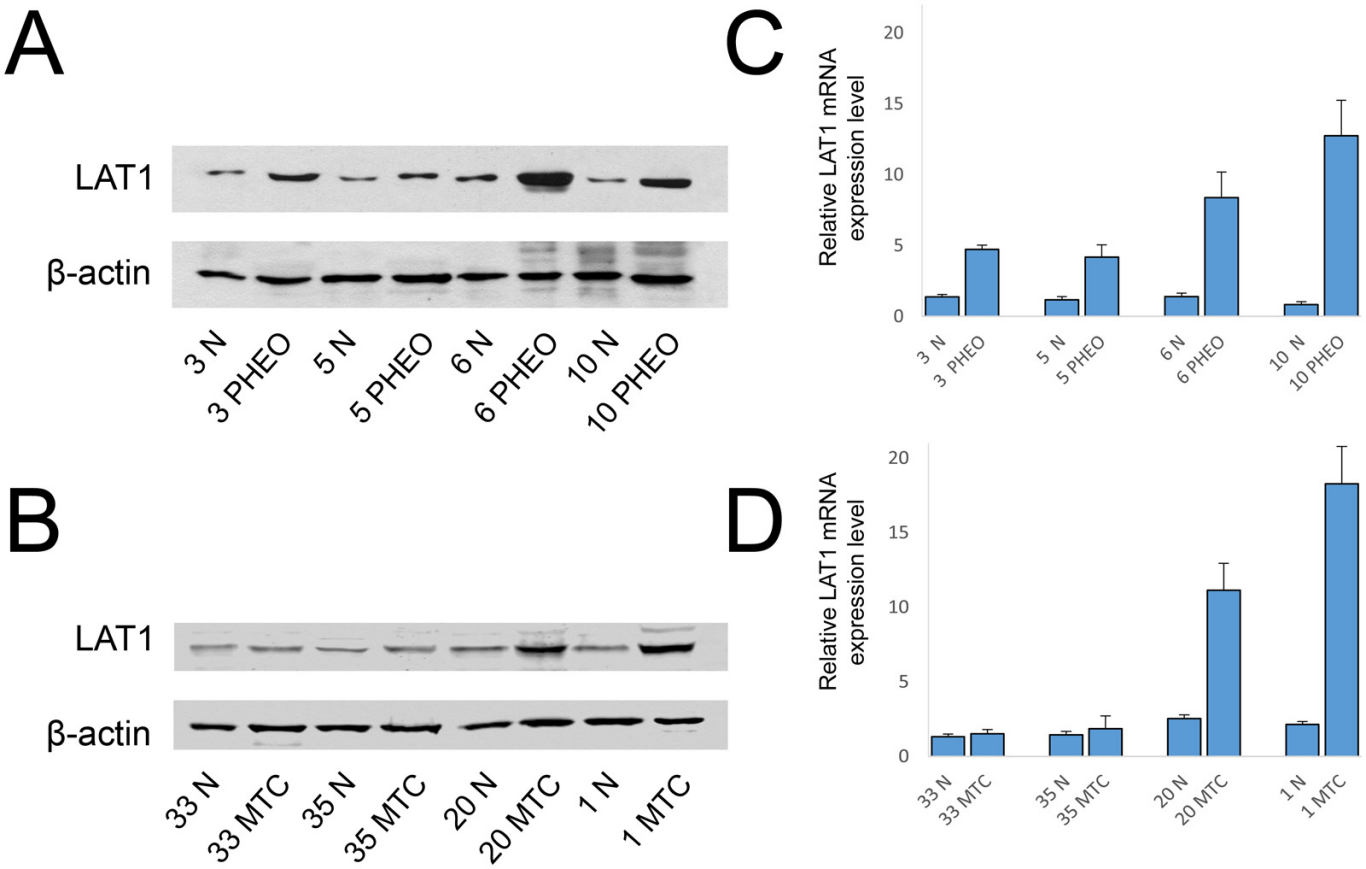
Western blot analysis and corresponding *LAT1* mRNA expression level. Representative Western blot analysis of normal (N)/tumoral match-pair samples for the expression of LAT1 in PHEO (**A**) and MTC (**B**) samples. Samples were corrected for protein loading by β-actin. Corresponding bar graphs for relative mRNA expression level of *LAT1* in (C) PHEO and MTC (D).

We then focused on LAT1 because it was expressed more strongly. Western blot experiments testing LAT1 expression in four representative normal/tumoral PHEO paired samples confirmed that the tumoral part expressed more protein than the non-tumoral tissue (Fig 2A). A stronger expression of LAT1 in tumors than in normal tissues was thus confirmed, and this trend was the same that we found in the mRNA expression analysis (Fig 2C).

### *LAT1*, *LAT2* and *GLUT1* gene expression in MTC

**Fig 3:**
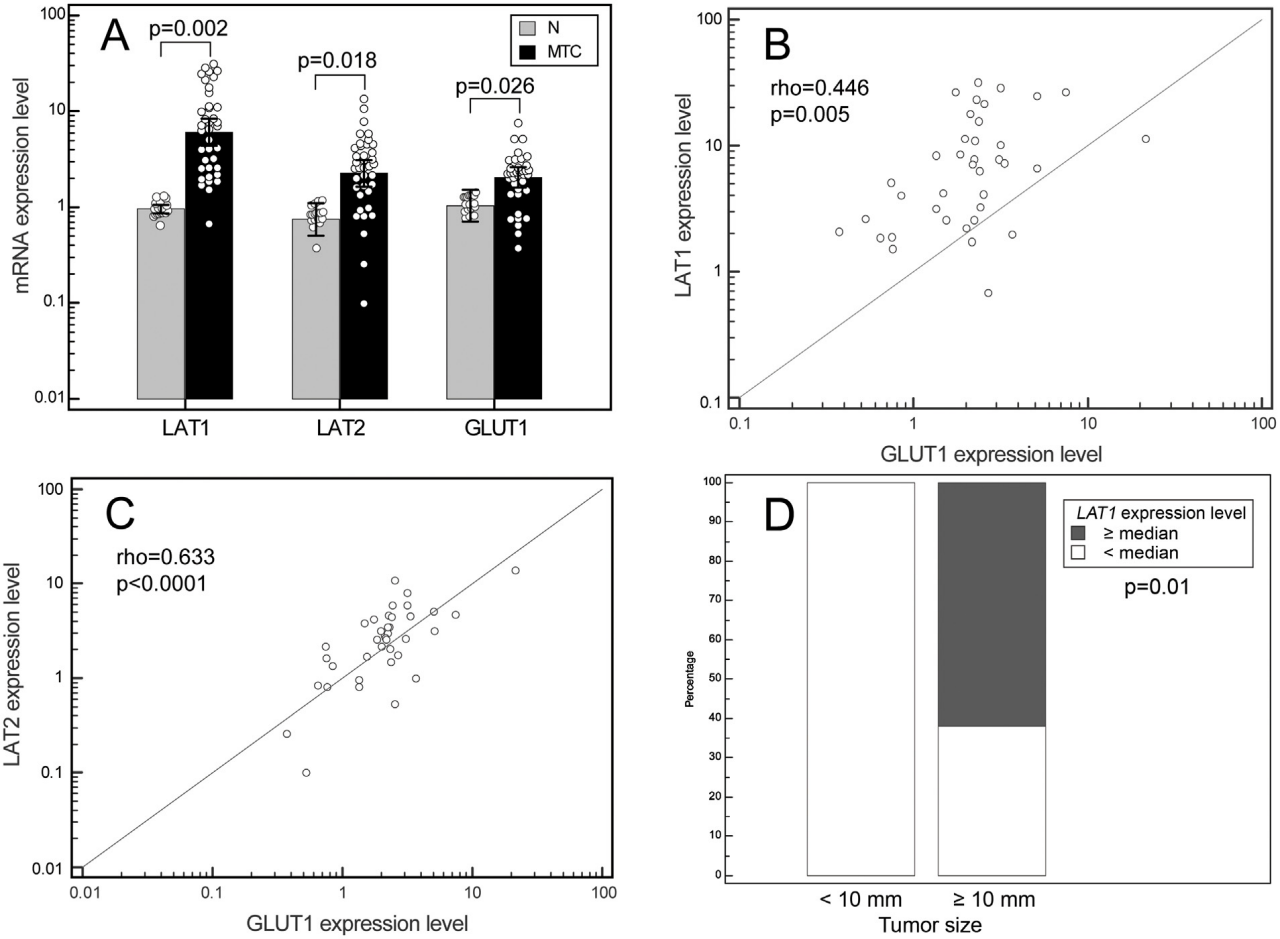
LAT1, LAT2 and GLUT1 genes expression in MTC. (A) Box plots of relative qPCR gene expression measurements of *LAT1*, *LAT2* and *GLUT1* in MTC specimens and the relative paired normal tissues. Each value was referred to a pool of normal thyroid tissues that was set to 1. Each dot represents a single sample. (**B**) and (**C**) Associations between the *LAT1* and *GLUT1* (**B**) and the *LAT2* and *GLUT1* (**C**) gene expressions in MTC specimens, by Spearman’s rank correlation. (**D**) Frequency chart representing the Fisher’s exact test correlation between the LAT1 expression levels in MTC, dichotomized according to the median value, with the tumor size dichotomized for the size of 10 millimeters.

MTC specimens showed a significant increase in *LAT1* (p = 0.002), *LAT2* (p = 0.018) and *GLUT1* (p = 0.026) mRNA levels by comparison with normal thyroid tissues. In particular, their upregulation was 9.93-fold for *LAT1* (95% CI: 6.61–12.45), 4.11-fold for *LAT2* (95% CI: 2.33–4.16), and 2.65-fold for *GLUT1* (95% CI: 1.69–3.94) (Fig 3A). Here again, *LAT1* transcription is more strongly enhanced than *LAT2* mRNA (p = 0.0002), while no such differences were seen in normal thyroid tissues.

Spearman’s rank correlation coefficients were calculated to investigate the associations between *LAT1*, *LAT2* and *GLUT1* gene expressions in MTC. A positive relationship between *LAT1* and *GLUT1* expression (rho = 0.446, p = 0.005) (Fig 3B) and a strong positive relationship between *LAT2* and *GLUT1* expression (rho = 0.633, p<0.0001) were found (Fig 3C).

The differences in mRNA expression levels were then considered in relation to clinical aspects: only tumor size greater than 10 mm correlated with a higher than median *LAT1* expression level (p = 0.01, Fig 3D). Patients’ clinical characteristics are summarized in Table 2.

**Table 2:** Clinical features of patients with medullary thyroid cancer.

*N°*	*Gender*	*Age at diagnosis (years)*	*CT pre (ng/L)*	*Size (mm)*	Stage*	*Status*	^†^*LAT1 expression level*
1	F	45	537	33	II	cured	high
2	F	62	20.4	8	III	cured	low
3	F	49	267	18	I	cured	high
4	F	45	83	10	I	persistent	high
5**	F	27	940	15	I	persistent	low
6	M	56	3720	24	II	persistent	high
7	M	55	200	47	II	persistent	high
8	F	61	438	12	III	persistent	low
9	M	59	6410	4	IV	persistent	low
10	M	73	2310	55	IV	persistent	high
11	M	70	52.9	20	IV	persistent	high
12	M	41	1600	11	IV	persistent	high
13	M	64	629	15	IV	persistent	high
14	F	37	22.68	20	III	persistent	high
15	M	45	532	24	IV	persistent	high
16	M	81	700	42	IV	persistent	low
17	F	74	n.a.	n.a.	n.a.	persistent	low
18	M	66	2000	26	IV	persistent	low
19	M	60	22931	40	IV	persistent	low
20	M	59	n.a.	n.a.	IV	persistent	high
21	F	64	19000	30	IV	persistent	high
22	M	67	17260	30	IV	persistent	low
23	M	52	8800	25	IV	persistent	high
24**	F	10	18860	20	IV	persistent	low
25	M	22	n.a.	n.a.	IV	persistent	low
26	F	77	991	18	I	cured	low
27	F	37	442	18	I	cured	high
28	F	73	163	8	I	cured	low
29	M	54	1136	20	I	cured	high
30** ^¶^	F	36	76.9	6	I	cured	low
31	F	71	62.2	7	I	cured	low
32	F	54	347	33	II	cured	high
33	F	74	700	50	III	cured	low
34	F	61	2258	30	III	cured	high
35	F	73	n.a.	14	III	cured	low
36	M	64	950	14	IV	cured	high
37	F	61	413	26	n.a.	cured	low
38	M	n.a.	n.a.	n.a.	n.a.	cured	low

Abbreviations: F, female; M, male; n.a., not available.

*7^th^ TNM classification;

**familiar MTC,

^¶^MEN2 patient where PHEO and MTC were analyzed simultaneously for LAT1 mRNA expression level (see results section).

^†^ LAT1 mRNA expression divided into high and low levels according to the median value.

Western blot experiments testing LAT1 expression in four representative paired normal*/*tumoral MTC samples showed that the tumoral part expressed more protein than the non-tumoral tissue in the most part of assessed cases (Fig 2B) showing that the expression at mRNA level reflects the amount of LAT1 protein (Fig 2D).

In one MEN2A patient, we were able to analyze both PHEO and MTC tumor tissues simultaneously for the three genes: for PHEO, *LAT1* expression was 41 times higher than in normal tissues, while *LAT2* and *GLUT1* genes were expressed on much the same levels as in normal tissue; for MTC, on the other hand, *LAT1* expression was 6.5 times higher while that of *LAT2* was 3.1 times and that of *GLUT1* was 5 times higher than in normal thyroids.

### LAT1 immunohistochemistry

**Fig 4:**
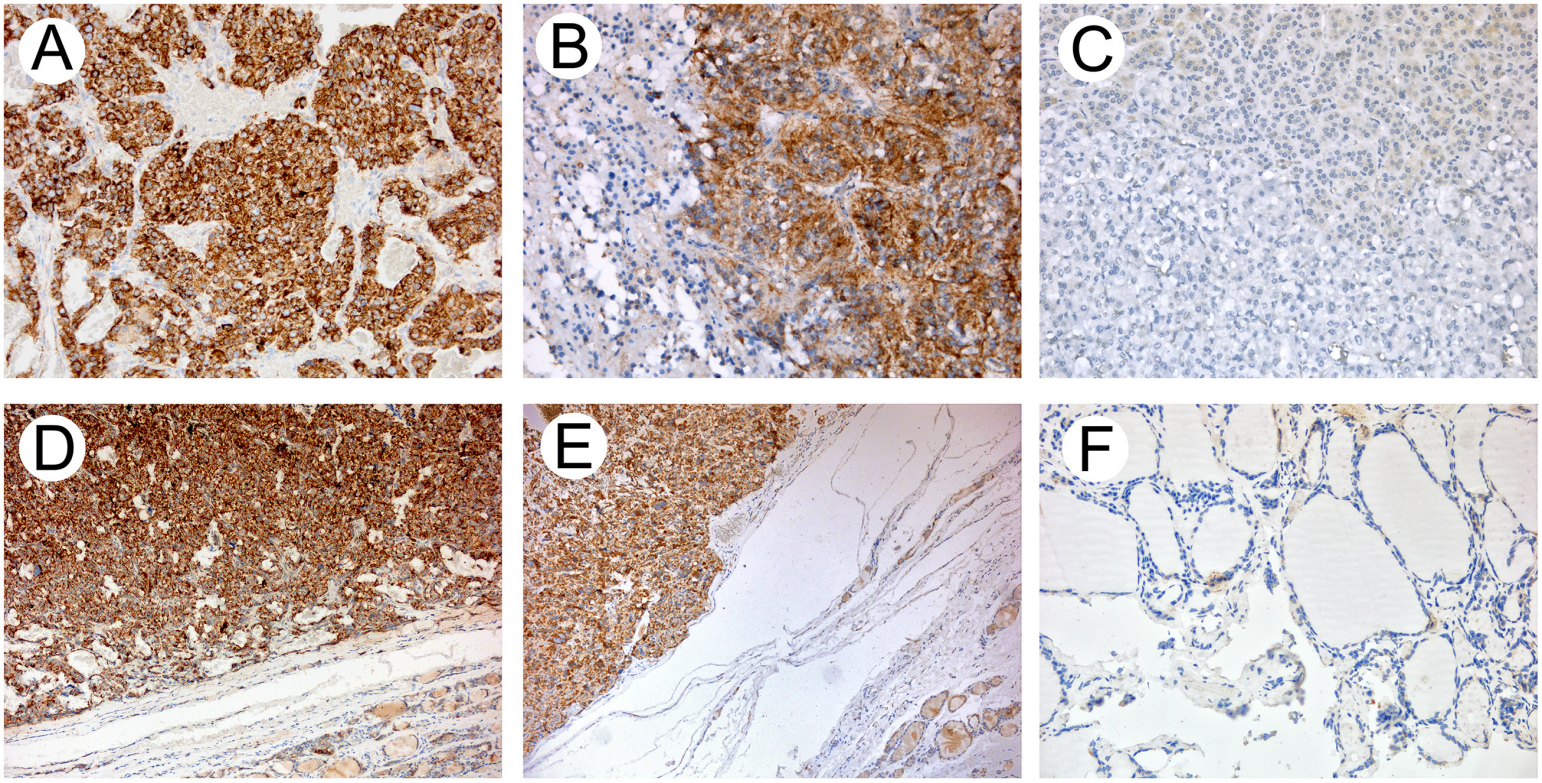
LAT1 immunohistochemistry. (**A**-**B**) Pheochromocytoma, strong cytoplasmic staining of LAT1 with membranous enhancement sparing of the neoplastic nuclei and normal adrenal tissue **(C)**. (**D-E**) Medullary thyroid carcinoma, strong cytoplasmic staining of LAT1 with membranous enhancement sparing of the neoplastic nuclei and normal thyroid tissue **(F)**. (**A-C and F**) 20× original magnification; (**D-E**) 10× original magnification.

LAT1 protein expression was then assessed in paired normal/tumoral PHEO and MTC samples using immunohistochemistry (IHC). As previously shown in other tissues [16], LAT1 protein again displayed a strong cytoplasmic expression with membranous enhancement, in both PHEO and MTC, by comparison with normal tissues in all assessed tissues (Fig 4). The LAT1 reactivity was comparable with the Western blot findings for tumoral samples investigated and mainly revealed a cytoplasmic distribution.

## Discussion

While initially developed for the neurological scanning of the basal ganglia in Parkinson’s disease and in patients with psychiatric disorders, ^18^F-FDOPA was shown to accumulate in PHEO, MTC, and gastroenteropancreatic NET—due to such tumors’ ability to take up, store, and decarboxylate L-DOPA. Applications of this metabolic tracer in PET/CT scanning have proved more effective than anatomical imaging methods not only for the purpose of identifying the tumor burden (particularly in patients with recurrent disease), but also in providing further functional evidence to support patient prognosis and treatment planning.

In metastatic PHEO, several studies have demonstrated that the diagnostic accuracy of ^18^F-FDOPA-PET is clearly superior to that of ^123^I-MIBG or CT/MRI, and that ^18^F-FDOPA-PET can also be performed in the presence of drugs interfering with MIBG uptake [17–19]. ^18^F-FDOPA scanning is now considered the first-line imaging tool for detecting glomus tumors at diagnosis or localizing extra-adrenal PHEO, and in patients with hereditary PHEO syndromes [20,21].

It has been also demonstrated that ^18^F-FDOPA PET/CT produces better results than other functional/anatomical imaging procedures in patients with persistent or recurrent MTC, regional metastases, and basal calcitonin over 60 pg/ml, in which case scanning with ^18^F-FDOPA seems to be the most useful functional imaging method for detecting regional lymph node disease and identifying candidates for surgery with a curative intent [20,22].

While it has been demonstrated that VMAT1 protein expression is the molecular prerequisite for functional imaging of PHEO with ^123^I-MIBG scintigraphy [20], no experimental data on the molecular mechanism responsible for ^18^F-FDOPA uptake have been published to date. To our knowledge, this is the first study in the literature to analyze the levels of expression (both mRNA and protein) of the two main LAT isoforms (LAT1 and LAT2) responsible for ^18^F-FDOPA uptake in PHEO and MTC. The expression of these two transporters was also correlated with that of *GLUT1* at mRNA level.

Using real-time PCR, we demonstrated that *LAT1* and *LAT2* are overexpressed in both PHEO and MTC samples by comparison with normal adrenal medulla and thyroid tissue. *LAT1* was markedly more overexpressed than *LAT2*, and more so in PHEO (with 15.1- and 4.1-fold increases, respectively) than in MTC (with 9.9- and 4.1-fold increases, respectively). To further validate our results concerning *LAT1* mRNA expression, we ran parallel Western blot experiments to test LAT1 protein expression in normal/tumoral samples: we demonstrated a positive trend between mRNA levels and protein expression, confirming that the difference in LAT1 expression between PHEO/MTC and their normal counterparts emerges at protein level too.

These data were also confirmed with immunohistochemical analysis; in fact LAT1 protein showed a strong cytoplasmic expression with membranous enhancement, in both PHEO and MTC, by comparison with normal tissues. There are many evidences in literature that LAT1 immunostaining, especially in tumor tissue, is not only on plasma membrane, as we can expect about a transporter protein, but also diffuse in cytoplasm and/or in granules within the cytoplasm. Considering that after translation on ribosomes the membrane proteins are packaged into transport vesicles in the cytosolic compartment, we can suppose that the overexpression of LAT1 in the tumors involves an accumulation of vesicles in the cytoplasm. However, there were described some cases in which neoplastic cell showed only a strong cytoplasmic positivity [23].

Concerning PHEO patients, the statistically significant link between their *LAT1* overexpression and their high levels of urinary catecholamines strongly suggests that this amino acid transporter may be a leading molecular step not only in the higher ^18^F-FDOPA uptake, but also in the increased catecholamine secretion. However no significant correlation between *LAT2* expression levels and the urinary levels of catecholamine was found. This may depend on at least two causes: the first, the amount of LAT1 is higher than that of LAT2 in cancer cells as shown in Shennan et al. 2003 [24]; additionally LAT1 transports large neutral amino acids with higher affinity than LAT2 [7].

*LAT1* has recently been associated closely with cancerous and/or proliferative cells, and previous studies found it strongly expressed in proliferating tissues, in many tumor cell lines, and in primary human tumors [25,26]. From a basic oncological viewpoint, cancer cells need plenty of nourishment for rapid growth and cell division, and *LAT1* expression has been described as a significant indicator of a poor outcome in various human cancers, including lung [27], pancreas [28] or breast [29], and hepatocellular cancer [30]. Cancer cell growth is also supported by an increased glucose metabolism: this phenomenon corresponds to an increased glucose uptake across the plasma membrane by the glucose transporter proteins (GLUT). Several studies have demonstrated that the expression of glucose transporters, and of GLUT1 in particular, increases in a variety of malignancies. *GLUT1* overexpression has also been found associated with tumor progression and poor overall survival in various malignant tumors [31]. From a clinical viewpoint, previous findings showed that ^18^F-FDG-PET (which visualizes glucose metabolism and glucose transport, mainly via the GLUT1 transporter) performs better than other functional imaging procedures with more specific tracers such as ^18^F-FDOPA, particularly in cases of malignant PHEO, and especially in patients carrying *SDHB* mutations. Our molecular PHEO findings reveal some degree of discrepancy on this issue, since we demonstrated a good correlation between *LAT1/2* and *GLUT1* expression levels, but the levels of the latter were comparable in tumoral and normal adrenal medulla cells. Such apparently conflicting data might be explained by the fact that the majority of PHEO patients in our series had benign disease; only 2 cases showed a malignant behavior according to the presence of distant metastases; finally, none of them carried SDHB mutations.

Our MTC patients revealed a good correlation between *LAT1/2* and *GLUT1* expression levels, together with an increase in *GLUT1* gene expression, though less marked than that of ^18^F-FDOPA transporters. This confirms that functional imaging with ^18^F-FDG PET could have a complementary role in the radiological work-up of a subset of MTC patients, particularly those with recurrent disease and low calcitonin doubling times, as a surrogate of aggressive disease, as recently suggested [32].

As concerns tumor size, we demonstrated a correlation between *LAT1* overexpression and tumors larger than 1 cm in size, but only in MTC, not in PHEO. The presence of necrotic tissue and degenerative rearrangement (a frequent finding in larger PHEO) can cause variations in tumor size, and might explain the lack of correlation in PHEO, compared to MTC finding.

In conclusion, the present study is the first to provide experimental evidence of the overexpression in some NET cancers (such as PHEO or MTC) of L-type amino acid transporters, and the *LAT1* isoform in particular, giving the molecular basis to explain the increase of the DOPA uptake seen in such tumor cells. Further studies are needed to define if the LAT1/LAT2 overexpression is also the molecular basis for justifying the ^18^F-FDOPA use in functional imaging of NET cancers.

## Supporting Information

S1 Fig Western blot analysis for LAT1 antibody validation. Western blot showing the LAT1 protein expression (apparent molecular weight of 38 kDa, red bands) and the β-actin loading control (apparent molecular weight of 42 kDa, green bands), detected by LI-COR infrared fluorescence IRDye^®^ secondary antibodies. LAT1 ABcam antibody validation analysis in two cell lines (**A**): the first cell line (H1299 lymph node metastasis of the lung cells) does not express LAT1 and has therefore been used as a negative control; the second cell line (MCF7 breast cancer cells) over express LAT1 and has therefore been used as a positive control. Whole picture Western blot of normal (N)/tumoral match-pair samples for the expression of LAT1 in PHEO (**B**) and MTC (**C**) samples. SHSY5Y cells (human neuroblastoma) and TT cells (human MTC) were inserted as tumor positive control of the considered disease.
(TIF)
