# Supplementary figures and images for: Overexpression of L-Type Amino Acid Transporter 1 (LAT1) and 2 (LAT2): Novel Markers of Neuroendocrine Tumors
Source: PLoS One. 2016 May 25;11(5):e0156044. doi: 10.1371/journal.pone.0156044 (PMC4880303; doi:10.1371/journal.pone.0156044)

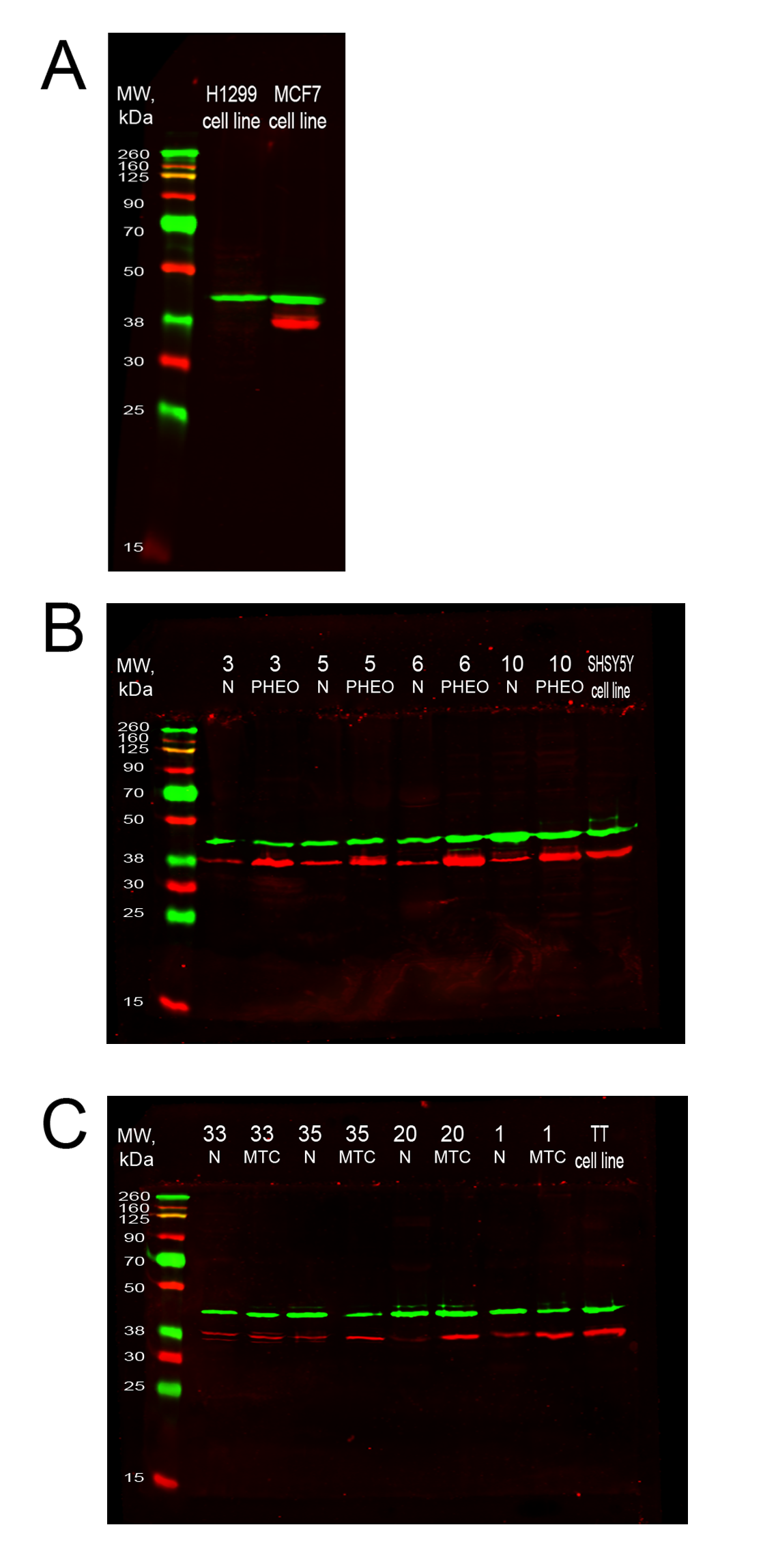

Supplement: S1 Fig — Western blot showing the LAT1 protein expression (apparent molecular weight of 38 kDa, red bands) and the β-actin loading control (apparent molecular weight of 42 kDa, green bands), detected by LI-COR infrared fluorescence IRDye® secondary antibodies. LAT1 ABcam antibody validation analysis in two cell lines (A): the first cell line (H1299 lymph node metastasis of the lung cells) does not express LAT1 and has therefore been used as a negative control; the second cell line (MCF7 breast cancer cells) over express LAT1 and has therefore been used as a positive control. Whole picture Western blot of normal (N)/tumoral match-pair samples for the expression of LAT1 in PHEO (B) and MTC (C) samples. SHSY5Y cells (human neuroblastoma) and TT cells (human MTC) were inserted as tumor positive control of the considered disease. (TIF) [file pone.0156044.s001.tif]
